# Biomarkers of Aging: From Cellular Senescence to Age-Associated Diseases

**DOI:** 10.1155/2017/7280690

**Published:** 2017-08-02

**Authors:** Maria João Martins, Miguel Constância, Delminda Neves, Andreas Simm

**Affiliations:** ^1^Institute for Research and Innovation in Health (i3s), University of Porto, 4200-135 Porto, Portugal; ^2^Department of Biomedicine, Unit of Biochemistry, Faculty of Medicine of the University of Porto (FMUP), 4200-319 Porto, Portugal; ^3^Department of Obstetrics and Gynaecology, University of Cambridge, Cambridge CB2 0SW, UK; ^4^Department of Biomedicine, Unit of Experimental Biology, Faculty of Medicine of the University of Porto (FMUP), 4200-319 Porto, Portugal; ^5^Heart Centre of the University Hospital Halle (Saale) of the Martin-Luther-University Halle-Wittenberg, 06120 Halle (Saale), Germany

The major common risk factor of degenerative diseases is the age of the person. For a long time, the calendrical age was used to calculate the risk to develop a disease or to have a negative outcome. Indeed, calculated by the Gompertz survival function, mortality rate increases exponentially with time. Older patients have a higher risk to get a worse outcome in comparison to younger patients. The plasticity of stem cells is reduced with age. Whereas all these data seem to fit on the basis of cohorts, on the level of individuals, age, as calculated time, is a bad predictor. We all know fit people at the age of 80 and old people at the age of 70. Therefore, one needs a better calculation of the age of a person, the so-called biological age. This age should be analysed using biomarkers of aging. In contrast to the calendrical age, the biological age can be influenced by the environment and our behaviour. The collection of review and primary research papers in this special issue of *Oxidative Medicine and Cellular Longevity* covers a wide range of topics from mechanisms of cellular aging to nutritional and lifestyle interventions in aging and age-associated conditions.

The article by J. Strycharz et al. is an in-depth review of the role played by the pleiotropic P53 gene in metabolic homeostasis regulation and age-associated obesity and diabetes. The authors provide insights into p53-dependent metabolic actions in the adipose tissue, liver, pancreas, and muscle and discuss how p53 activation may contribute to the regulation of insulin resistance across the life course. The papers by S.-T. Oh et al. and J. Mikuła-Pietrasik et al. are focused on vascular endothelial dysfunction, a pathology that is commonly observed in age-related cardiovascular and brain diseases. These studies demonstrate that native low-density lipoprotein (LDL) or serum from varicose patients induces premature senescence of endothelial cells (HUVECs), with both treatments leading to the generation of reactive oxygen species (ROS). In the case of LDL, the downstream mechanisms involve modulation of p53, p21, and p16-pRb signal transduction pathways after LDL receptor-mediated endocytosis of native LDL. In the case of serum from varicose patients, the production of ROS is TGF*β*1-dependent. Importantly, this study proposes that varicosity generates local (endothelium-related) and systemic (serum-related) proinflammatory conditions, leading to “inflammaging.” Still on the theme of cellular senescence, L. Matos et al. demonstrate that copper-induced fibroblast senescence can be attenuated by resveratrol through positive modulation of protein quality control systems (namely, autophagy upregulation) and, consequently, improvement of cellular proteostasis. Â. Carneiro and J. P. Andrade argue, in a compelling review paper, that lifestyle changes, including nutritional advice, may delay the onset of age-related macular degeneration (where increased oxidative stress and/or endothelial dysfunction are relevant) and can slow the progression of the disease. Finally and also on lifestyle choices, M. Tolahunase et al. consider in their paper whether yoga and meditation (YMLI) can impact on cellular aging. They show that YMLI reduce the rate of cellular aging, as measured by improvements in both cardinal (DNA damage, telomere attrition, and oxidative stress) and metabotropic (neuroplasticity, stress and inflammatory responses, and longevity) blood biomarkers of cellular aging.

Aging is a major risk factor and the basis for degenerative diseases. Any efforts to reduce the risk for developing those conditions will require a better understanding of the cellular and molecular mechanisms of aging and their effects on homeostasis regulation. Overall, the articles in this series describe common molecular targets involved in cellular aging and provide insights into the plasticity of the aging process that can be “modulated” and potentially “delayed” or “reversed.” Much more research is needed to identify how precisely “delaying” or “reversing” aging will impact on the metabolic disease burden. In this regard, a particular interesting avenue of future research is epigenetic reprogramming, since one of the hallmarks of aging is epigenetic drift. In times of rapid dietary and environmental change, understanding the cross-talk between the environment and genome, through epigenetics, is likely to be important not only for the understanding of the aging process and its relationship with degenerative disease risk but also for the immediate health of human populations through the design of therapeutic-targeted approaches.

